# Immunoglobulin G is a natural oxytocin carrier which modulates oxytocin receptor signaling: relevance to aggressive behavior in humans

**DOI:** 10.1007/s44192-023-00048-z

**Published:** 2023-10-19

**Authors:** Henning Værøy, Emilie Lahaye, Christophe Dubessy, Magalie Benard, Marion Nicol, Yamina Cherifi, Saloua Takhlidjt, Jean-Luc do Rego, Jean-Claude do Rego, Nicolas Chartrel, Sergueï O. Fetissov

**Affiliations:** 1 Department of Psychiatric Research, Akershus University Hospital, 1478 Nordbyhagen, Norway; 2 INSERM 1239, Neuroendocrine, Endocrine and Germinal Differentiation and Communication Laboratory, University of Rouen Normandie, 76000 Rouen, France; 3 INSERM US51, CNRS UAR 2026, Imagine Platform PRIMACEN- HeRacLeS, Institute for Research and Innovation in Biomedicine (IRIB), University of Rouen Normandie, 76000 Rouen, France; 4 INSERM US51, CNRS UAR 2026, Behavioral Analysis Platform SCAC-HeRacLeS, Institute for Research and Innovation in Biomedicine (IRIB), University of Rouen Normandie, 76000 Rouen, France

**Keywords:** Aggressive behavior, Neuroendocrinology, Neuropeptides, Oxytocin, Oxytocin receptor, Intracellular signaling, Autoantibodies, Human, Mice, Brain, c-fos

## Abstract

Oxytocin is a neuropeptide produced mainly in the hypothalamus and secreted in the CNS and blood. In the brain, it plays a major role in promoting social interactions. Here we show that in human plasma about 60% of oxytocin is naturally bound to IgG which modulates oxytocin receptor signaling. Further, we found that IgG of violent aggressive inmates were characterized by lower affinity for oxytocin, causing decreased oxytocin carrier capacity and reduced receptor activation as compared to men from the general population. Moreover, peripheral administration of oxytocin together with human oxytocin-reactive IgG to resident mice in a resident-intruder test, reduced c-fos activation in several brain regions involved in the regulation of aggressive/defensive behavior correlating with the attack number and duration. We conclude that IgG is a natural oxytocin carrier protein modulating oxytocin receptor signaling which can be relevant to the biological mechanisms of aggressive behavior.

**Supplementary Information:**

The online version contains supplementary material available at 10.1007/s44192-023-00048-z.

## Main text

Defensive/aggressive behavior belongs to the basic motivated behaviors set to preserve the organism at both an individual and a community level. Nevertheless, aggressive behavior manifesting in some individuals as increased hostility and violence, remains a major challenge for the modern society and requires a better understanding of its biological origin. Several molecular targets, neuronal and neuroendocrine pathways have been implicated in the regulation of defensive/aggressive behavior [1–4]. For instance, oxytocin (OT), a 9 amino acids regulatory peptide produced mainly in the hypothalamus and secreted into the bloodstream and in various regions of the CNS, is known for playing a major role in promoting social interactions and for reducing stress and aggression [5–7]. Importantly, OT may modulate social, aggressive and other types of stress-related behavior by acting on the OT receptors (OT-R) expressed in several brain regions and also in the peripheral nervous and olfactory systems, as well as in the adrenal gland [8–12]. Whereas reduced plasma OT was reported in aggressive subjects [13], OT supplementation in humans has not consistently yielded desired prosocial effects, pointing to a more complex regulation of oxytocin signaling relevant to aggressive behavior [14–16].

The immune system impacts on the brain function with a major role of proinflammatory cytokines, as a part of the innate immune response to infection, interfering with mood and behavior, including aggression [17, 18]. Adaptive immunity may also play a role in the regulation of stress-response and motivated behavior. For instance, adrenocorticotropin (ACTH)-reactive IgG autoantibodies (autoAbs) modulate ACTH-induced cortisol secretion in both aggressive and non-aggressive subjects [19]. OT-reactive autoAbs have also been detected in humans correlating with some psychopathological traits, ex. in depression [20]. Moreover, elevated levels of OT-reactive autoAbs were found in subjects with conduct disorder [21]. However, the functional relevance of OT-reactive autoAbs to OT signaling and to aggressive behavior remained unknown. Here we show that OT in plasma circulates bound to IgG, which plays a role of OT carrier protein modulating OT signaling at the OT-R and that such IgG may change c-fos activation in the brain associated with aggressive/defensive behavior in mice. We further show that OT-reactive IgG in men who had committed acts of severe violence underlie decreased oxytocin carrier capacity and reduced OT-R activation.

In this study, both the OT peptide and OT-reactive IgG(s) were analyzed in men with a history of severe violence by including subjects from a high security prison in Norway who were sentenced for crimes involving violent acts such as murder. The Inmates’ group (n = 16) is hereafter named Aggressive (Aggr) or “Aggressors”. For comparison, a control group (Contr, n = 19) of men from the general population in Norway was included. This study was approved by the National Research Ethics Committee, case number 2010/792 and was performed in accordance with the ethical standards as laid down in the 1964 Declaration of Helsinki and its later amendments or comparable ethical standards. Informed consents were obtained from all participants. The studied groups did not significantly differ for age, (45 *vs.* 42 years-old) or body mass index (BMI, 29.70 ± 3.24 *vs.* 26.2 ± 3.55 kg/m^2^), inmates *vs.* controls, respectively. Degree of aggressive behavior was evaluated by the self-administered Bryant and Smith revised Aggression Questionnaire (BS-rAQ) [22]. A highly significant increase of the total BS-rAQ score as well as its subscales of “physical aggression”, “hostility” and “anger” characterized the aggressive group (Suppl. Table 1).

**Fig. 1:**
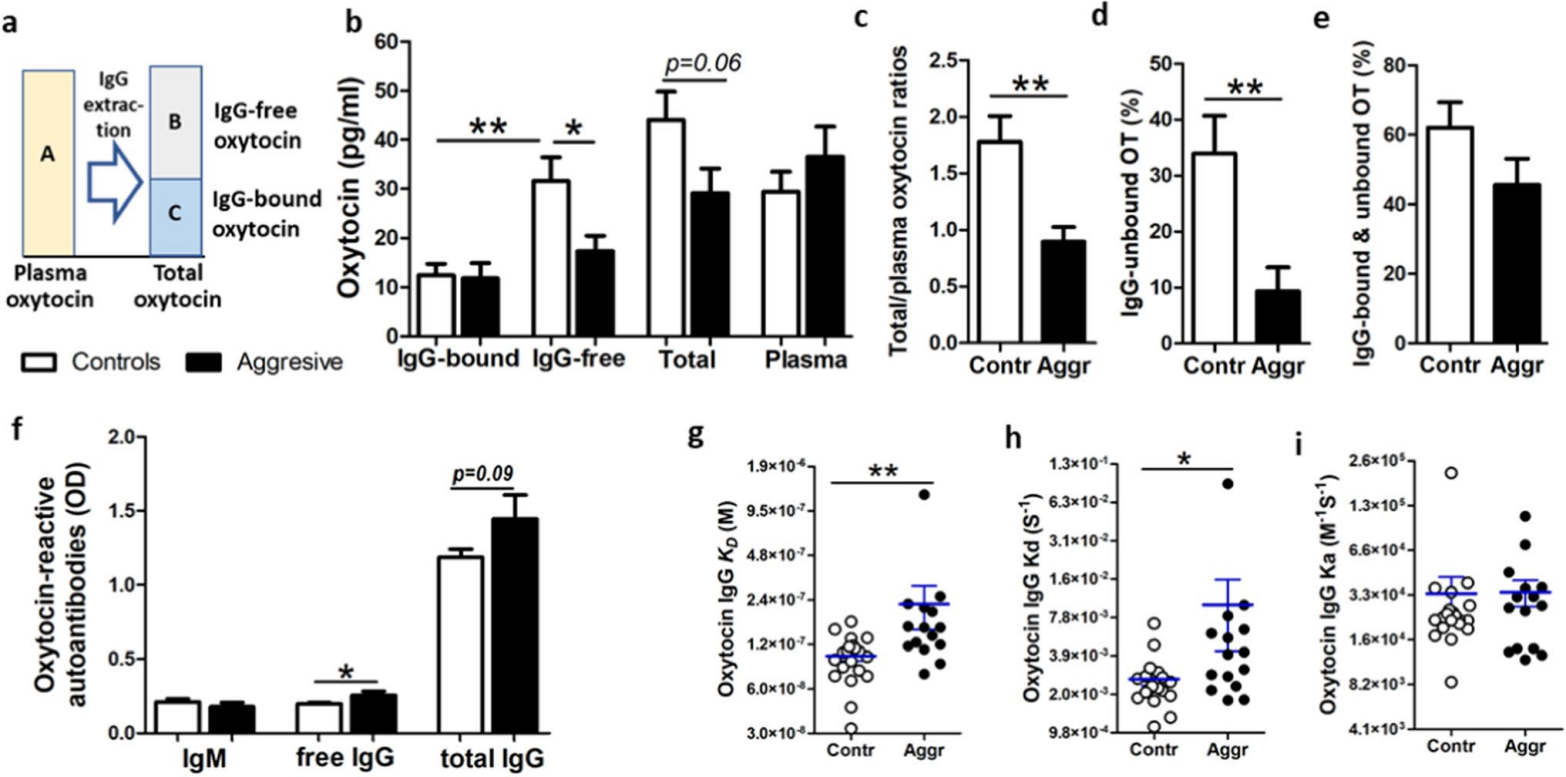
OT peptide and OT-reactive immunoglobulins. OT concentrations assayed in different OT fractions labeled by capital letters in (**a**), including native plasma (**A**), IgG free (**B**) and bound OT (C) as well as total OT (A + B) are shown in (**b**). Estimation of IgG OT carrier capacity by analyzing total/plasma OT ratios (**c**) and IgG unbound OT (**d**). Percentage of IgG bound and unbound OT (**e**). Plasma levels of OT-reactive IgM and IgG (**f**). Affinity kinetics of IgG for OT including the dissociation equilibrium constant, K_D_ (**g**) and the rates of association, K_a_ (**h**) and dissociation, K_d_ (**i**). **b**, **c**, **f,** Student’s t-test, **d**, **g**, **i**, Mann–Whitney test, *p < 0.05, **p < 0.01

Previous studies showed that OT in plasma is largely bound to proteins, but their nature has remained unknown [23, 24]. To determine if IgG may play a role of an OT-carrier, we studied if OT can be detected unbound from IgG after IgG chromatographic extraction from plasma (for methodology see Additional file 1: Fig. S1). Further, to analyze how much of OT circulates bound to IgG, we measured OT levels in both IgG-free and IgG-bound fractions and compared them with the OT levels measured in native plasma, *i.e.* before IgG extraction (Fig. 1a). We found that in all study subjects, OT was detectable at the picomolar levels in both IgG-bound and IgG-free fractions (Fig. 1b). However, in the Control but not the Aggressive group, the OT levels were higher in IgG-free than in IgG-bound fractions (Fig. 1b). Moreover, IgG-free OT levels were higher in Controls vs. Aggressors and the total OT levels, *i.e.* a sum of IgG -bound and -free OT, also tended to be higher in Controls, while the mean OT levels measured in native plasma did not differ between the groups (Fig. 1b). We have also noticed that in Controls the mean levels of total OT were slightly higher than OT levels in native plasma samples. Indeed, by analyzing their ratios, they were found to be higher in the Control group (Fig. 1c). These results indicate that when assayed in native plasma, some OT escapes detection, probably because it was washed away bound to IgG during peptide extraction from plasma by C18 columns, representing one of the common challenges for the accurate OT plasma assay [25]. In contrast, when an earlier step of IgG purification is introduced, such OT can be unbound from IgG, contributing to higher levels of IgG-free OT found in the Control group. Indeed, the percentage of IgG-unbound OT was higher in Controls (Fig. 1d). Moreover, a sum of IgG-bound and unbound OT tended to be higher in Controls than in Aggressors accounting for an average of 62% and 45% from the total OT, respectively (Student’s t-test p = 0.13, Fig. 1e). Thus, these data reveal that OT is naturally and reversibly bound to plasmatic IgG supporting its role as an OT carrier protein and that the carrier capacities of IgG for OT are reduced in the Aggressive group.

To characterize the properties of OT-reactive Ig, we analyzed their levels and affinities. No significant differences in OT-reactive IgM levels were found between the groups (Student’s t-test p = 0.3, Fig. 1f). However, levels of OT-reactive free IgG, *i.e.* assayed in normal buffer, were higher in Aggressors (Fig. 1f) while the levels of total OT-reactive IgG, *i.e.* measured in a stringent buffer to dissociate IgG peptide immune complexes (IC), tended to be higher (Student’s t-test p = 0.09). A relative increase in IgG, but not IgM, autoAbs levels points to the absence of a recent OT-like antigenic stimulation in aggressors. Such possibility is further supported by analyzing affinity kinetic parameters using surface plasmon resonance. Indeed, higher values of the dissociation equilibrium constant (KD), *i.e.* decreased affinity of OT-reactive IgG, were found in the Aggressors’ group (Fig. 1g). The KD changes were mainly due to an increased dissociation rate (Kd, Fig. 1h), since the mean values of the association rate (Ka) did not differ (Mann–Whitney test p = 0.45, Fig. 1i). Thus, a decreased micromolar affinity of IgG for OT in aggressors confirms our suspicion that such IgG are less efficient to carry OT. Of relevance, increased micromolar affinity of peptide hormone-binding IgG may also underlie its better protection from enzymatic degradation [26].

**Fig. 2:**
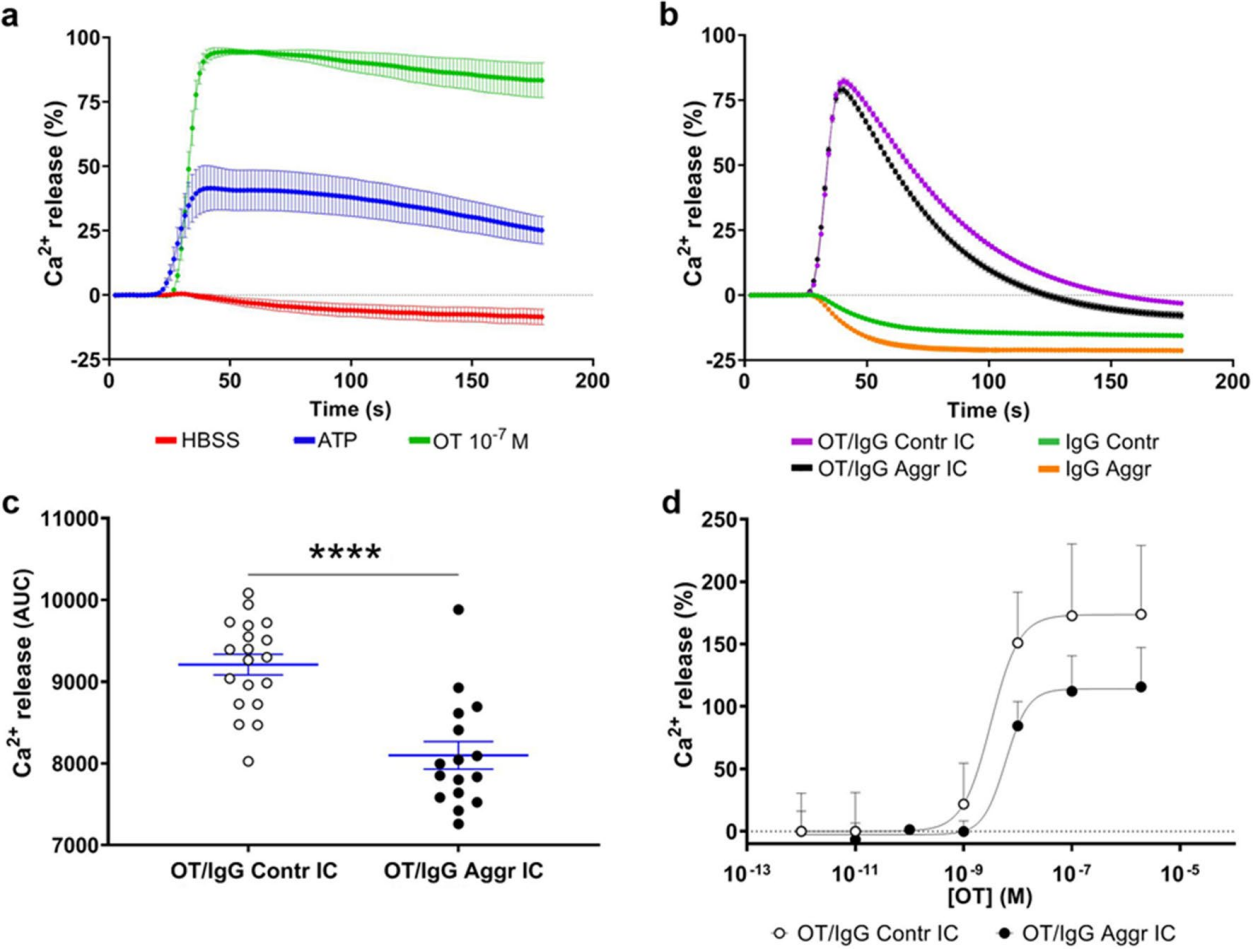
Activation of human OT-R in HEK293 cells in vitro. **a** Stimulation of intracellular Ca^2+^ mobilization using OT (10^−7^M), as well as adenosine triphosphate (ATP, 10^−4^M) and HBSS buffer as positive and negative controls, respectively. **b** Intracellular Ca^2+^ mobilization by OT/IgG IC from the aggressor or control groups or with IgG alone. **c** Comparison of OT/IgG IC-induced total Ca^2+^ release between aggressors and control groups using the area under curve (AUC). **d** OT dose–response for OT/IgG IC-induced of Ca^2+^ release in OTR-expressing cells. **c** ****p < 0.0001 Student’s t-test

Stimulation of the intracellular release of Ca^2^^+^ is considered to be the main signal transduction pathway for the OT-R activation [27]. To study whether OT/IgG IC are able to stimulate OT-R-mediated intracellular Ca^2+^ mobilization, we used human embryonic kidney (HEK) 293 cells, stably transfected with human OT-R [28]. The capacity of these cells to mobilize Ca^2+^ was confirmed by ATP inducing a mild increase of Ca^2+^ release (Fig. 2a). Application of OT (10^−7^M) resulted in a strong (approximately 3.5-fold over basal levels) intracellular increase of Ca^2+^, confirming the specificity of the OT-R activation (Fig. 2a). For the in vitro formation of IC, individual IgG purified from plasma samples of inmates or controls (840 nM) were preincubated overnight with OT (2 × 10^−6^M). Application of OT/IgG IC resulted in an immediate increase of intracellular Ca^2+^ similar to that observed after OT alone, albeit with about 20% lower peak levels (Fig. 2b *vs.* 2a). The dynamic of Ca^2+^ mobilization was, however, quite distinct, with a more transient increase induced by OT/IgG IC in contrast to the long-lasting plateau after OT alone (Fig. 2b *vs.* 2a). Such modified dynamics of Ca^2+^ release was observed after incubation with OT/IgG IC from either inmates or controls (Fig. 2S). However, the statistical comparison of the OT-R activation profiles, revealed lower mean levels of total Ca^2+^ mobilization when IgG from the aggressive subjects were used (Fig. 2c). Although the differences in Ca^2+^ peak values did not reach significance between the groups (Student t-test, p = 0.09), the time of the peak appearance and of the return baseline were shorter in the aggressive group (data not shown, Mann–Whitney tests, both p < 0.001). Incubation of OT-R expressing cells with IgG alone did not activate the intracellular Ca^2+^ mobilization, confirming that it required presence of OT (Fig. 2b). Effective concentrations (EC50) of OT/IgG IC for activation of the OT-R were found in the nanomolar range and were higher in aggressors *vs*. controls (6.1 ± 0.2 × 10^–9^ M *vs*. 3.1 ± 0.8 × 10^−9^M, Student’s t-test p < 0.05) when assayed at fixed IgG concentration of 840 nM (Fig. 2d). A nanomolar range of EC50 values for the OT peptide (1.4 × 10^−9^M) has been reported for activation of OT-R in the same cellular assay [28]. Thus, OT/IgG IC activate OT-R almost as efficient as the OT peptide alone with IgG playing a modulatory role.

**Fig. 3:**
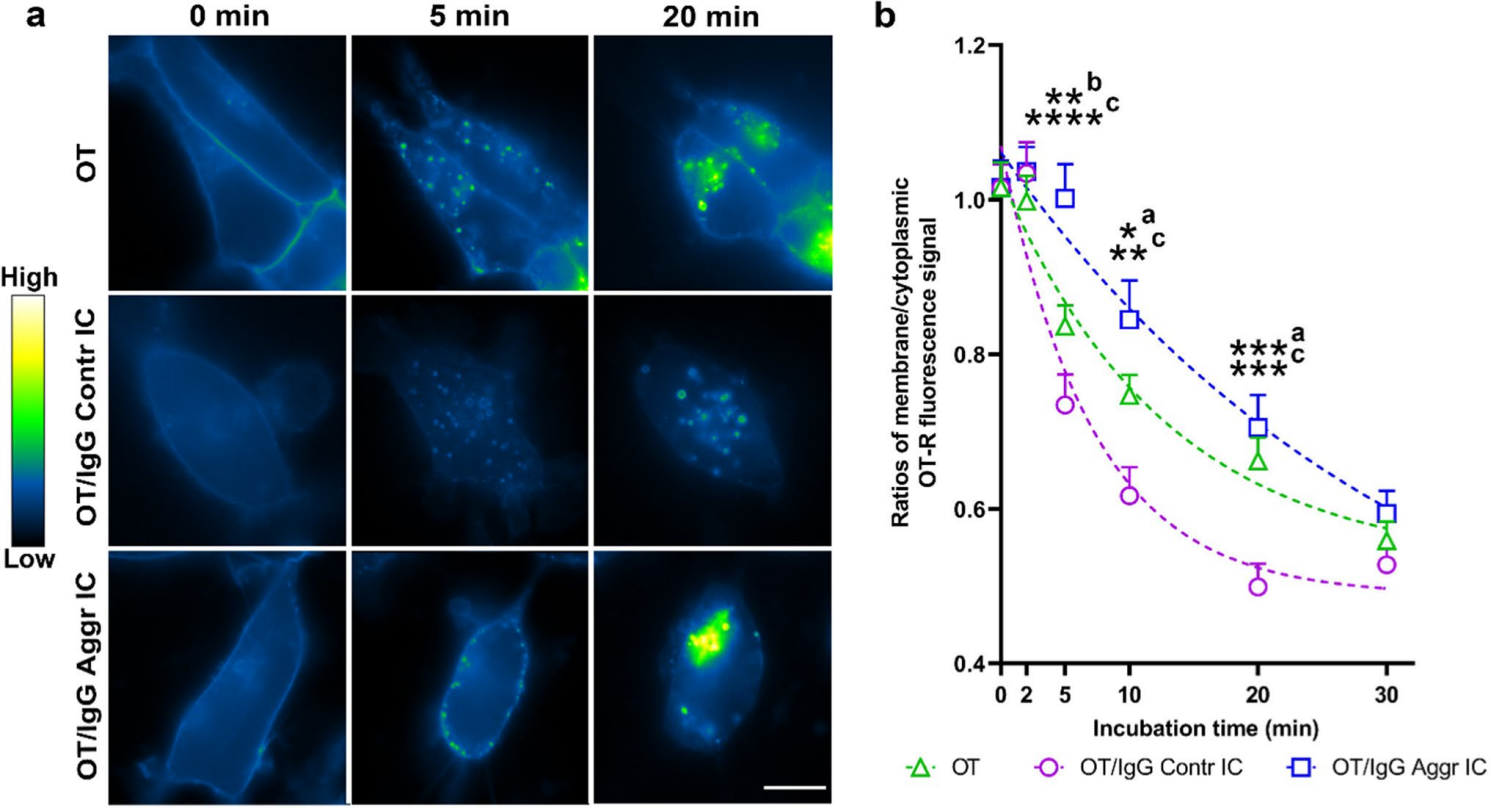
Dynamics of GFP-labeled human OT-R cellular internalization in vitro. **a** Upper line, OT alone, 2nd line OT/IgG Contr IC, and 3rd line OT/IgG Aggr IC. Vertical columns correspond to the time-points of microscopy- before (0 min), and 5 or 20 min after application of OT alone or OT/IgG IC. Pseudocolored fluorescence signal corresponds to the GFP-labeled human OT-R which relative intensity level is shown by a color-map. Scale bar 10 μm. **b** OT-R internalization rates were quantified by ratios between membrane and cytoplasmic OT-R fluorescence, dashed lines reflect the data fit by exponential decay model. Two-way repeated measurement ANOVA p < 0.0001, Bonferroni post-tests *p < 0.05, **p < 0.01, ***p < 0.001 and ****p < 0.0001, **a** OT *vs.* OT + IgG Contr, **b** OT *vs.* OT + IgG Aggr, **c**, OT + IgG Contr *vs*. OT + IgG Aggr

To further study the mechanisms of the OT-R activation by OT/IgG IC, we evaluated the in vitro dynamics of OT-R cellular internalization using video microscopy of green-fluorescence protein (GFP)-labeled human OT-R expressing HEK293 cells [29]. Before application of test ligands, diffuse fluorescent OT-R signal was visible along the cell membrane (Fig. 3a). Starting from 5 min after application of OT or OT/IgG IC, the membrane-associated fluorescence became scattered, while numerous fluorescent vesicles appeared in the cytoplasm (Fig. 3a). Representative images for all time-points are available in Supplementary materials (Fig. 3S). Quantification of the membrane/cytoplasmic fluorescent signals showed that their ratios were equal at time 0, followed by their more pronounced decrease in the Contr *vs*. Aggr groups at 5, 10 and 20 min of incubation (Fig. 3b). Applying the exponential decay model (Fig. 3b, dashed lines) revealed that the half-time internalization rate of OT-R was 16.7 ± 2.5 min for OT, 6.4 ± 0.7 min for OT/IgG Contr IC, and 23.3 ± 3.2 min for OT/IgG Aggr IC (Kruskal–Wallis test, p = 0.0002, Dunn’s post-test Aggr *vs*. Contr, p = 0.0001). Since internalization rates reflect receptor activation upon ligand binding, the obtained results point to the modulatory role of IgG in this process. Especially, a lower rate OT-R internalization by OT IC with IgG from Aggressors corroborates our results on Ca^2+^ signaling. Taken together, the in vitro data reveal a direct physiological modulation of OT-R signaling by the OT/IgG IC.

**Fig. 4:**
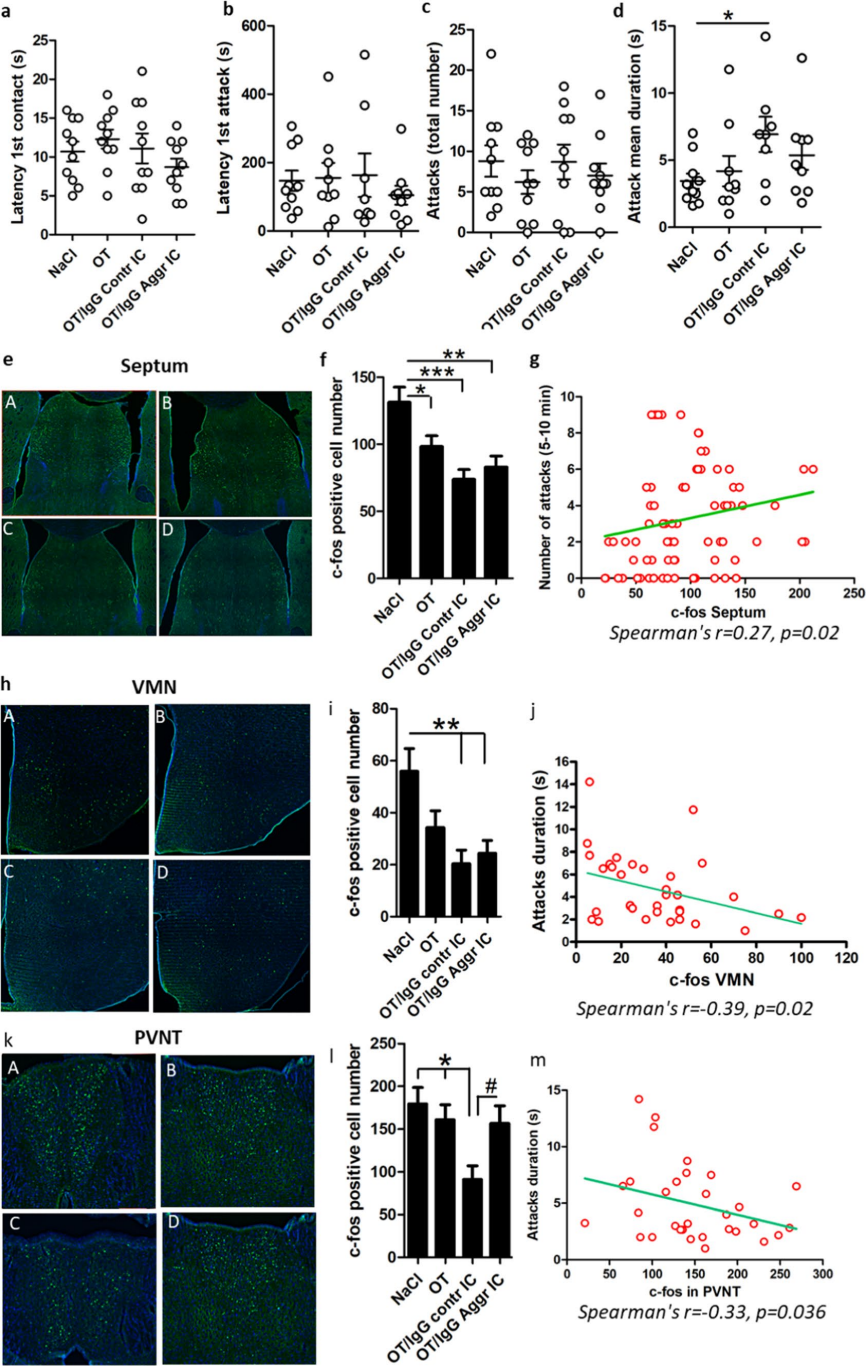
Resident intruder test (RIT) and brain c-fos immunohistochemistry in mice. **a** First contact latency. **b** First attack latency. **c**. Number of attacks. **d** Individual attack duration. Immunohistochemical detection of c-fos protein (green) in the brain after the RIT in the septum (**e**), ventromedial nucleus of the hypothalamus (VMN, **h**) and the paraventricular nucleus of the thalamus (PVNT, **k**), each panel subdivided in 4 images corresponding to the groups of 0.9% NaCl (A), OT (B), OT/IgG Contr IC (C) and OT/IgG Aggr IC (**D**). Quantification of c-fos positive cells in the septum (**f**) VMN (**i**) and PVNT (**l**). Significant Spearman’s correlations between c-fos-cell number and behavior are shown in **g**, **j** and **m**, in the septum (**g**) for number of attacks, and in VMN (**j**) and PVNT (**m**) for attack duration. Student’s t-test *p < 0.05 (**d**), ANOVA p < 0.0001 (**f**), p = 0.003 (**i**) and p = 0.009 (**I**), Tukey’s post-tests *p < 0.05, **p < 0.01 and ***p < 0.001, Student’s t-test #p < 0.05 (**l**). c-fos number (**f**, **i**, **l**) was calculated bi-laterally, n = 20

**Fig. 5:**
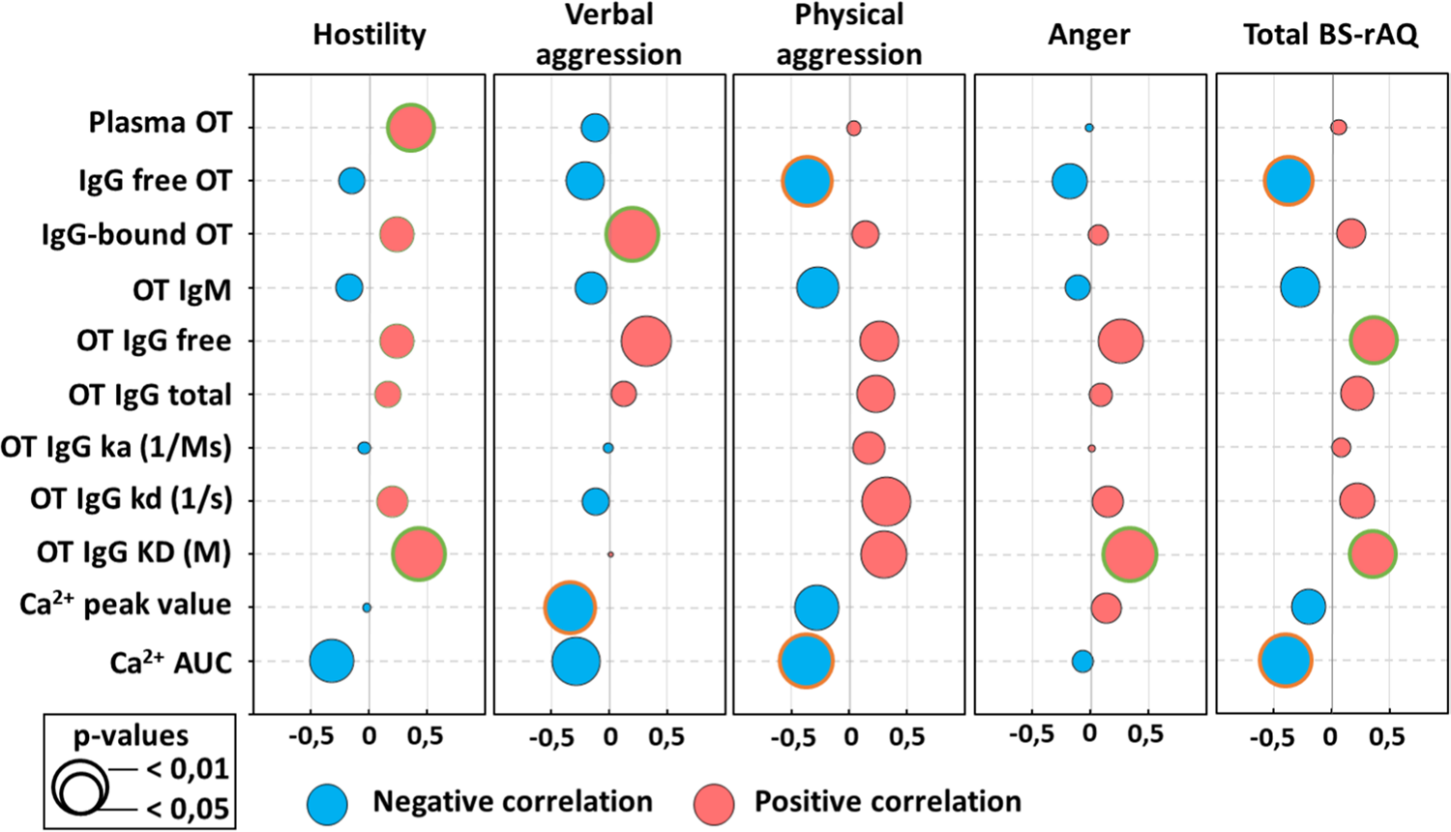
Correlations of the BS-rAQ aggressivity scores with OT signalling-related biomarkers. Correlation values (Pearson’s r) are illustrated by the diameter of the bubble and direction by colour for negative (blue) and positive (red) correlations. Surrounding colour indicates significant differences as specified in the legend. For exact p-values see the Supplementary Table 2

To validate the functional implication of OT/IgG IC in the modulation of aggressive/defensive behavior, we used a mouse model of resident/intruder test (RIT) [30]. Four hours prior to the RIT, resident mice were injected intraperitoneally with 0.9% NaCl or OT alone or with OT forming IC with a pool of OT-reactive IgG affinity-purified from plasma samples of Aggressors and Controls (n = 10 in each group). This protocol was selected based on previous data showing the presence of IgG in the brain parenchyma starting from 4h after their peripheral administration [31]. Behavioral analysis revealed no-significant effect of treatment on the latency of the first contact and first attack or the total number of attacks (Fig. 4a–c). However, each attack duration was increased in mice receiving OT with IgG from Controls pointing to an increased defensive behavior in this group. The immunohistochemical analysis of c-fos expression in the brain of resident mice revealed numerous c-fos positive cells in regions involved in regulation of defensive/aggressive behavior including the septum (Fig. 4e), the ventromedial nucleus of the hypothalamus (VMN, Fig. 4h) and the anterior paraventricular nucleus of the thalamus (PVNT, Fig. 4k), for a higher resolution figures see Supplementary materials (Figs. 4, 5 and 6S). Mice treated with OT/IgG IC from Aggressive and Control groups both showed lower c-fos-positive cell number in the septum and VMN as compared to 0.9% NaCl-treated mice, while the OT only injected mice showed intermediate results (Fig. 4f, i). In the PVNT, a decrease of c-fos-positive cells was observed only in mice receiving OT/IgG IC from Controls. (Fig. 4l). The number of c-fos cells correlated negatively with each attack duration in the VMN and PVNT (Fig. 4j, m) but positively with attack number in the septum (Fig. 4g). Thus, the results of this animal experiment revealed that peripheral administration of OT-reactive human IgG modulate defensive/aggressive behavior and the brain c-fos response to the RIT.

Finally, we analyzed associations between OT-related biological parameters with psychological measures of aggressiveness as reflected by the BS-rAQ scores. As expected, the total BS-rAQ score, which was increased in aggressive subjects, correlated positively with OT-IgG levels and their KD values but negatively with free/unbound OT levels and OT/IgG IC-induced Ca^2+^ mobilization (Fig. 5 and Suppl. Table 2). In addition to these correlations which suggest pro-social effects of increased OT signaling, the OT peptide levels in native plasma correlated positively with “Hostility” scores. Of interest, a previous study reported increased hostility in humans receiving intranasal OT [15]. Taken together, these results further support a role of both OT and OT-binding IgG in a continuum of different modalities of aggressive behavior in humans associated with their either decrease or increase, the latter linked to OT- binding IgG levels and properties.

The reasons for individual and group differences in OT-reactive IgG levels and affinities found in this study are presently unknown and in theory, may involve both genetic and environmental factors including infection agents. Of interest, significant associations between childhood infections and violent behavior in adulthood have been reported, while early developmental OT changes are known to shape up regulation of social interactions in later life [32, 33]. A link between gut microbiota composition and both central and peripheral OT signaling was also suggested [34, 35]. Whatever the antigenic origin of OT-binding IgG, the present study has established that they play a role of an OT carrier protein in the systemic circulation modulating this hormone’s activation of the OT receptor. This role of IgG appears as a natural phenomenon *i.e.* underlying physiological OT-signaling. Although our study does not provide a direct evidence that OT/IgG IC were present in the brain, we showed that their peripheral administration is perceived by the septum and VMN, brain regions well-known for their role in regulation of defensive/aggressive behavior [16, 36]. The PVNT also has recently attracted attention as a key component of the brain circuits regulating reward, motivational conflict and defensive behavior which can be mediated via the OT-R [37–39]. The possibility of a direct effect of OT/IgG IC on central OT-R is supported by previous studies showing the physiological presence of peripherally injected IgG in the brain parenchyma [31]. Moreover, it was recently found that the soluble receptor for advanced glycation end-products (RAGE), a 50 kDa protein of the Ig superfamily, can bind OT and facilitates its uptake into the brain [40]. These data further support the potential functional role of circulating Ig in OT transport across the blood–brain barrier.

Peripheral administration of OT/IgG IC in mice consistently reduced the number of c-fos in the brain. These result most likely reflect enhanced activation of the OT-R down-stream pathways and reduction of perceived stress during the RIT [41]. In this context, it should be noted that in rodents OT is not always reduces aggression but can be also adaptively pro-aggressive depending on the behavioral context, sex and phenotype [16, 42]. For instance, a significant reduction in the duration of attacks but not their frequency was observed among OT-KO mice [43]. Moreover, repeated intranasal OT administration in non-aggressive rats increased their aggressiveness [44]. Intranasal OT was also shown to reduce freezing behavior during the RIT in mice [41], supporting the idea of the contextual fear reducing effects of OT [45]. An increase of attack duration but not their number by OT/IgG, may appear as an example of such contextual modification of OT-induced defensive/aggressive behavior promoting active defensive behavior, while a decrease of the number of c-fos positive cells reflected the OT-mediated anti-stress effects. Thus, these results show that human OT-reactive IgG are able to modify aggressive behavior in mice in the RIT context. Nevertheless, since the mice data cannot be directly extrapolated to humans, we cannot be affirmative that the altered IgG properties to bind OT found in aggressive inmates may contribute to their aggressive traits.

To conclude, the study revealed that plasmatic IgG act as a functional carrier of the OT peptide in human plasma underlying individual variability of OT-R activation. Moreover, deficient carrier properties of IgG for OT associated with a reduced OT-R activation were found in prison inmates characterized by increased self-reported aggressive behavior scores. The relevance of this finding to aggressive behavior needs further investigation. For instance, the effect of incarceration on the IgG properties to bind OT should be analyzed in inmates with various degree of aggressiveness and compared with free subjects characterized by low and high aggression.

### Supplementary Information

Below is the link to the electronic supplementary material.

Supplementary file1:

## Data Availability

All data needed to obtain the conclusions in this study are present in the main paper or the supplementary materials and have been deposited at the Rouen University server.
