# Supplementary material for: Immunoglobulin G is a natural oxytocin carrier which modulates oxytocin receptor signaling: relevance to aggressive behavior in humans
Source: Discov Ment Health. 2023 Oct 19;3(1):21. doi: 10.1007/s44192-023-00048-z (PMC10587035; doi:10.1007/s44192-023-00048-z)
Supplement: Supplementary file 1 — Supplementary file1: [file 44192_2023_48_MOESM1_ESM.docx]

**Supplementary materials**

**Materials/Subjects and Methods**

*Study subjects*

This study was approved by the National Research Ethics Committee, case number 2010/792 and was performed in accordance with the ethical standards as laid down in the 1964 Declaration of Helsinki and its later amendments or comparable ethical standards. Informed consents were obtained from all participants.

*Violent aggressors*

16 violent aggressive inmates were included. 11 of these had committed at least one murder, or had attempted to commit murder, and 1 inmate had participated in gang-related activity resulting in murder. Four inmates had committed brutal physical violence with violent sex-related components, such as rape, molestation, or grievous bodily harm. Inmates who had committed paedophilic acts were excluded from this study.

All violent male inmates, except 1, were recruited from a high-security prison outside Oslo. The inmates were serving long-term sentences, the majority in preventive detention. One of the studied persons had been released and was tested between committing violent crimes. He was later re-arrested and charged following violent behaviour and is currently serving time in a different prison. In Norway, the imposition of preventive detention indicates that the court considers the defendant at high risk for reoffending, and therefore an imminent threat to society. According to Norwegian law, after having served a minimum term not exceeding 10 years, a prisoner in preventive detention may make an appeal to the court to reconsider his case. None of the 16 prisoners had serious mental illness.

*Non-aggressive subjects as the control group*

19 healthy male volunteers were included from various walks of life in Norwegian society. Healthy controls had no history of psychiatric disorders or ongoing psychiatric symptoms at the time of inclusion. They also had a clean criminal record and a regular job. Both controls and inmates underwent clinical psychiatric screening interviews to exclude past and present serious psychiatric (e.g. psychotic or bipolar disorders) and somatic conditions (e.g. serious head trauma and conditions of the nervous system). They were also examined for aggression levels as detailed below.

*The Aggression Questionnaire by Bryant and Smith (BS-rAQ)*

The original aggression questionnaire (AQ) published by Buss and Perry *(31)* had 4 scales: Physical Aggression, Verbal Aggression, Anger, and Hostility, which correlated differently with various personality traits. The scale scores were found to correlate with peer nominations of the various kinds of aggression, suggesting the need to assess individual aggressiveness components. Bryant and Smith (19), later found that the 4 scales did not show adequate common variance (i.e. about 80%), and they consequently omitted items with low loadings or multiple loadings, and excluded items with reverse-scored wording. This yielded a 12-item, refined four-factor measurement model, which not only contains fewer than half as many items as the original, but is also psychometrically superior. This refined 12-item aggression questionnaire was used in the present study to evaluate aggressiveness. BS-rAQ data from violent aggressors and healthy controls are shown in **Supplementary Table 1**.

*Blood samples*

After the screening interview, if the subject was eligible, venous blood samples were collected in EDTA tubes from a cubital vein, stored on ice before centrifugation, after which plasma was drawn and the sample stored at -80°C until transported on dry ice and then thawed for oxytocin and IgG analyses as described below. The same samples were used for the assay of oxytocin peptide and IgG.

*Free and bound oxytocin plasma assay*

Oxytocin concentrations were assayed in native plasma samples as well as in the IgG bound and unbound plasma fractions. In all 3 cases, oxytocin enzyme-linked immunosorbent assay (ELISA) kit was used according to the manufacturer’s instructions (Enzo Life Sciences, Farmingdale, NY, USA). In brief, oxytocin from plasma samples was purified using C-18 and diluted in the “Assay buffer” provided in the kit and used for direct plasma OT assay. For the detection of “free”/IgG unbound oxytocin, plasma samples were incubated with Dynabeads Protein A (Thermo Fisher, Scientific, Waltham, MA, USA), the effluent was collected and used for oxytocin assay. Then, IgG were eluted from the beads using elution buffer (50 mM Glycine pH 2.8) and IgG bound oxytocin was measured. The OT carrier capacity of IgG was calculated as (OT free + OT bound)-OT plasma concentrations.

*Oxytocin-reactive autoantibody assays*

Plasma levels of oxytocin (OT)-reactive IgG and IgM were measured using ELISA according to a published protocol *(32)*. Briefly, oxytocin (Bachem AG, Bubendorf, Switzerland) was coated onto 96-well Maxisorp plates (Nunc, Thermo Fisher Scientific) using 100 µl and a concentration of 2 µg/ml in 0.5 M Na_2_CO_3_ and 0.5 M NaHCO_3_ buffer, pH 9.6 for 48 h at 4°C. The plates were washed (5 min x 3) in phosphate buffered saline (PBS); with 0.05% Tween 20, pH 7.4, and then incubated 3 hours at 37°C with 100 μl of human plasma diluted 1:400 in PBS to determine free OT-reactive IgG levels or in a dissociative 3 M NaCl, 1.5 M glycine buffer, pH 8.9 to determine total OT-reactive IgG levels. The plates were washed (3 x 5 min) and incubated for 3h with 100 μl of alkaline phosphatase (AP)-conjugated antibodies (1:2000, Merck, Darmstadt, Germany) at room temperature (RT). Following washing (3 x 5 min), 200 μl of p-nitrophenyl phosphate solution (Merck) was added as AP substrate. After 30 min of incubation at RT, the reaction was stopped by adding 3N NaOH. The optical density (OD) was determined at 405 nm using a microplate reader Infinite F200PRO (TECAN, Männedorf, Switzerland). Blank OD values resulting from the reading of plates without addition of plasma samples were subtracted from the sample OD values. Each determination was done in duplicate. The variation between duplicate values was less than 5%.

*IgG purification from plasma*

Total IgG were purified from plasma using Melon™ Gel Purification Kit (Thermo Fisher Scientific) according to the manufacturer’s instructions and a published protocol *(33)*. Briefly, Mini Spin Columns were loaded with 500 µL of Melon™ Gel purification support and centrifuged for 30 s at 5000 *g*. After two washes with Melon™ Gel purification buffer under the same centrifugation conditions, 500 µL of plasma (diluted 1:4 vol. in purification buffer) was incubated in the columns for 5 min at room temperature on a roller mixer. To collect purified IgG, Mini Spin Columns were centrifuged for 30 s at 5000 *g* and samples were lyophilized for 48 h, resuspended in HBS-EP buffer to 0,5mg/mL (Cytiva, Marlborough, MA, USA) for affinity kinetics analysis and other experiments and conserved at –80°C. Total IgG concentrations were evaluated using NanoDrop One (Thermo Fisher Scientific) with HBS-EP buffer as blanks.

*Affinity kinetics assay*

The affinity kinetics of plasma purified IgG for oxytocin was determined by surface plasmon resonance (SPR) using a BIAcore T200 instrument (Cytiva). Biotin-conjugated oxytocin peptide (Eurogentec, Seraing, Belgium) was diluted at 3nM in HBS-EP+ buffer, (Cytiva) and was covalently coupled on the sensor chips NA coated with streptavidin (Cytiva). All measures were performed on the same OT-coated chip. For the affinity kinetic analysis, a multi-cycle method was run with five serial dilutions of each IgG sample: 336, 168, 84, 42 and 21 (nmol) including a duplicate of 84 nmol and a blank sample (HBS-EP+ with NSB reducer buffer only). Each cycle included 2 min of analyte injection and 10 min of dissociation with flow speed 30 µl/min at 4^o^C. Between injections of each sample, the binding surface was regenerated with 10 mM glycine pH 2.5, resulting in the same baseline level of the sensorgram. The affinity kinetic data were analyzed using Biacore T200 Evaluation Software 3 (Cytiva). Langmuir’s 1:1 model was used to fit the kinetic data and the sample values were corrected by blank subtractions.

*In vitro oxytocin receptor activation assay*

HEK293 cells expressing human oxytocin receptors were generously provided by Prof Gerald Gimple from the Institute of Pharmacy and Biochemistry, Mainz, Germany *(24)*. Cells were grown on DMEM medium (Gibco, Thermo Fisher Scientific) with 10% FBS and 0.6% Penicillin/Streptomycin (Merck). To prepare assay plates, cell line was treated with 0.25% trypsin-EDTA (Gibco), cell pellet was resuspended in medium and filed in a flat clear bottom black 96-microwell plate (Greiner Bio-One, Les Ulis, France) at a rate of 50000 cells per well, which was coated during 1 h at 37°C with 100µg/mL of Poly-L-Lysin (Merck) and 1 h with 100µg/mL of Collagen I (Institut de Biotechnologie Jacques Boy, Reims, France). The microplate was incubated 24 hours at 37°C with 5% of CO_2_. In the meantime, 840 nM of purified IgG were resuspended in Hank’s Buffer Saline Solution (HBSS; Gibco) buffered with 5 mM HEPES (Gibco) and supplemented with 2.5 mM probenecid (Merck) with 2.10^-6^ M of oxytocin and incubated in a U-bottom 96-microwell plate (Greiner Bio-One) under slow agitation at 4°C overnight.

The OTR activation by immune complex was evaluated by intracellular calcium (Ca^2+^) release as described by Dubessy et al *(34)*. Briefly, confluent cells were incubated 45 min at 37°C with 2 µM Fluo-4 acetoxymethyl ester (AM) calcium dye (Life Technologies, Villebon-sur-Yvette, France) in complete HBSS, washed twice and incubated in 200 µL of the same complete medium at 37°C for 20 min. Fluorescence was recorded using a Flexstation III fluorescence microplate reader system (Molecular Devices, Saint-Grégoire, France) during 180 s with an excitation wavelength of 485 nm, an emission wavelength of 525 nm and a cutoff filter of 515 nm. After 17 s recording in basal conditions, 50 µL of immune complexes were injected by the system to assess their ability to induce calcium release.

After data smoothing and subtraction of mean fluorescence background from control wells without Fluo-4 AM, baseline was normalized, then reduced to 0 and values were normalized between 0 and 100%, the 100% value corresponding to the maximum fluorescence reached by oxytocin. Ca^2+^ releasing kinetics were determined, as well as area under curve, the maximal fluorescence peak and the time for which this maximum is reached. Fluorescence data were calculated as mean +/- SEM.

*In vitro oxytocin receptor internalization assay*

HEK293 cells expressing green-fluorescent protein (GFP) labelled human oxytocin receptors were generously provided by Dr. Bice Chini, Institute of Neuroscience, Milan, Italy *(25)*. Cell line were filed in MatTek’s 35mm Glass Bottom Dishes (MatTek, Ashland, MA, USA) treated with Poly-L-Lysin and Collagene I at a rate of 250000 cells per dish in 2 mL of medium and incubated 24 hours at 37°C with 5% of CO_2._ IC were prepared in sterile PBS with a final concentration of 1.0 μM of oxytocin and 0.5 µM of IgG pool of each group. The internalization was recorded with the video microscope Leica DMI 6000 TIRF (Leica, [Wetzlar, Germany](https://www.google.de/maps/place/Leica+Microsystems+GmbH/@50.5519715,8.4948705,17z/data=!3m1!4b1!4m2!3m1!1s0x47bc5ab9e333f871:0xdc2813dbad80d50d)), with a x63 magnification in an enclosure maintained at 37°C. 3 fields were observed and imaged 0, 2, 5, 10, 20 and 30 min after injection of 20 µl of IC. 45 cells were studied for each condition to determine maximum of membrane and cytoplasmic fluorescence with Fiji software in order to quantify oxytocin receptor internalization.

*Oxytocin-reactive IgG purification*

Purified total IgG from 10 controls and 10 aggressive subjects were pooled in Binding-Wash buffer (Tris-buffered-Saline with 0.1% T_20_) and were further purified using oxytocin-biotin with Pierce^TM^ Streptavidin Magnetic Beads (Thermo Fisher, Scientific, Waltham, MA, USA), according to manufacturer’s instructions. Briefly, 1 mg of magnetic beads were incubated with 20 µg of oxytocin-biotin conjugate overnight at 4°C under agitation. Beads were next incubated overnight under agitation 4 times with 6 mg of total IgG. Non-OT-reactive IgG were eliminated in the effluent fraction, while OT-reactive-IgG were eluted using Elution buffer (0.1M Glycine, pH2.0). Concentrations of purified OT-reactive IgG were measured using a NanoDrop 2000C (Thermo Fisher Scientific), and affinity for oxytocin of such OT-reactive IgG was assayed using Biacore T200 as detailed upper.

*Resident-intruder test*

Two-month old C57Bl6 male mice were purchased from Janvier Labs (L’Arbresle, France) and acclimated to the animal facility for 1 week with 12 h light-dark cycle, lights on at 7:00 a.m. Animal experiments were performed in accordance with the French and European Directives and the recommendation for care of laboratory animals (2007/526/EC) and the experiments were approved by the Regional Ethical Committee (Ref#8690-2017012614301658). Mice were fed *ad libitum* with standard pelleted rodent chow (2016 Teklad Global rodent diet, ENVIGO, USA) with drinking water always available. The resident-intruder test for evaluation of aggression was performed by introducing an intruder mouse into the home cage of the resident mouse *(28)*. Resident mice (n=40) were housed in isolation for 21 days without bedding change before testing. Intruder mice (n=12) were housed as a group of 4 mice per cage during 21 days. Resident mice were distributed into 4 groups (n=10, in each) and 4 h before the test received an intraperitoneal (IP) injection of either 0.1 ml of 0.9% NaCl as a control or the same volume of 0.9% NaCl with OT peptide alone (10^-6^ M), or with the same amount of OT peptide preincubated overnight with a pool of OT-reactive IgG (5.0 μg) purified from plasma of control or aggressive subjects as described above. The aggressive/defensive behavior of the resident mouse was assessed by measuring the latency before the 1^st^ attack, the total number of bite attacks and the duration of attacks during 10 min. The latency before the first non-aggressive contact was also recorded.

*Tissue preparation and Immunohistochemistry*

20 min after the Resident-intruder test, mice were anaesthetized by an (IP) injection of a lethal dose of Ketamine/Xylazine (3:1 vol, 0.1 ml) and perfused intracardially with 20 ml of PBS followed by 40 ml of 4% paraformaldehyde in PBS. Brains were dissected out and immersed in the same fixative overnight, then kept in PBS at 4% until cut using a vibratome (Leica VT1000S) to obtain 25 μm coronal sections from the septum to VMN. Floating sections were proceeded for immunohistochemical staining. Briefly, sections were permeabilized with PBS - Triton X100 0,3%/NaAzide 0,01% at 4°C under agitation during 24 h. Then, they were incubated with primary c-fos monoclonal antibodies (ab208942, 1/1000^e^) at 4°C during 48 h, followed by anti-mouse fluorescein-conjugated secondary antibody (Alexa Fluor 488 Donkey-anti-Mouse, 1/300^e^, Invitrogen A21202) for 2 h at RT, and counterstained with DAPI (Roche 10236276001, 2 µg/mL during 1 min). Sections were mounted on slides in an anti-fading agent (Sigma F8680) and coverslipped. After processing, sections were examined with a Thunder Tissue 3D fluorescence microscope (Leica) with objectives x10, x20. The number of c-fos positive cells in each brain region of interest were counted as a mean from 3 consecutive sections in each animal which was used to calculate the group differences (n=10 per group). For the illustration digital images were optimized for image resolution.

*Statistical analysis*

Data were analysed and graphs were plotted using the GraphPad Prism 5 & 9 (GraphPad Software Inc., San Diego, CA, USA). Normal distribution was evaluated by the Kolmogorov-Smirnov test. Group differences were analyzed by the analysis of variance (ANOVA) & or 2 ways with repeated measurements or by the non-parametric Kruskal-Wallis (K.W.) test with Tukey’s or Dunn’s post-tests, depending on the normality of data distribution. Where appropriate, individual groups were compared using two-sided Student’s t-test or the Mann-Whitney (M.W.) test depending on the normality. Internalization data were fitted with GraphPad Prism using a single-phase exponential decay model after removing of outliers by the ROUT method (Q=1%). Correlations were analyzed using the Pearson’s and Spearman’s tests.

*Data availability*

All the data are available upon request.

**Supplementary figures
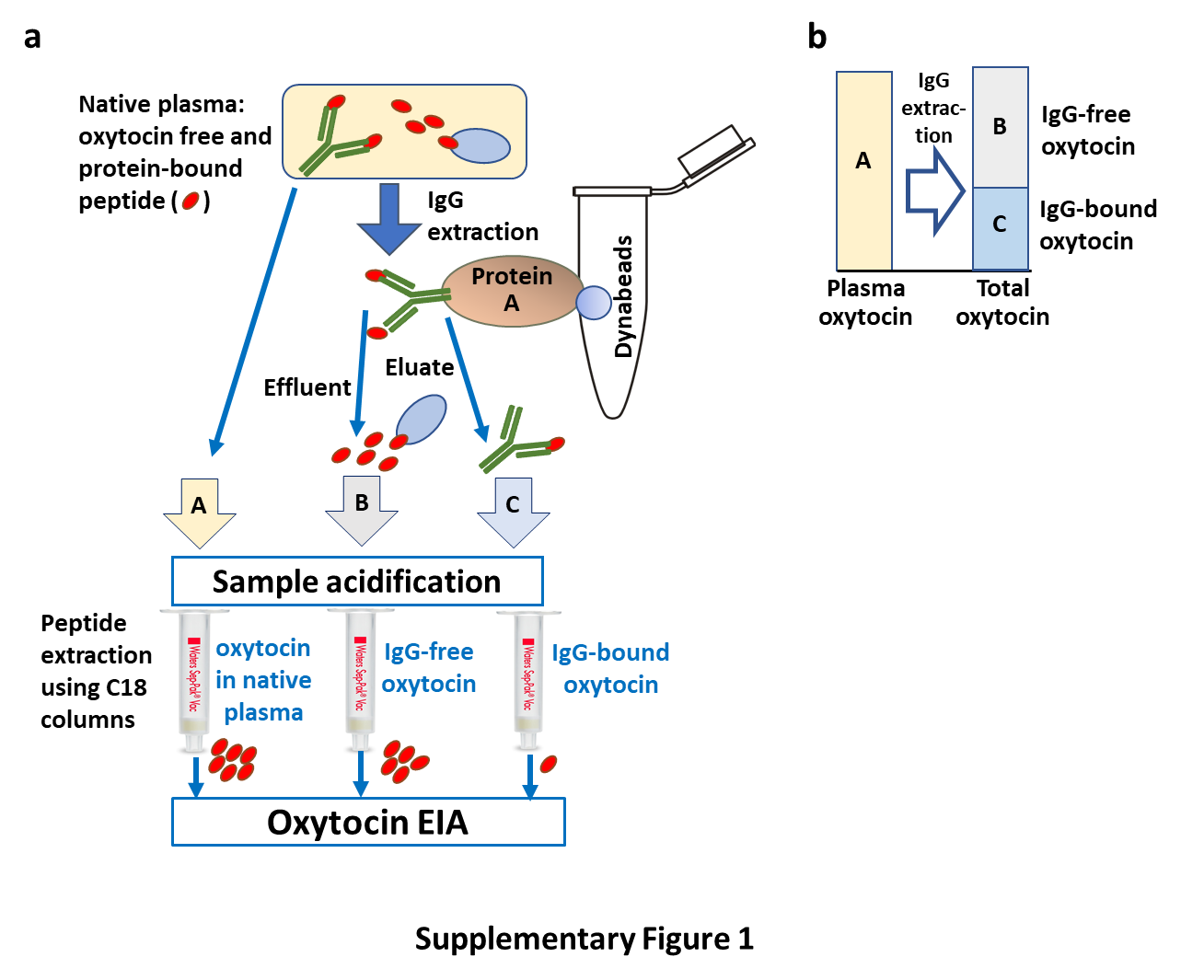
**

**Supplementary Figure 1S. a.** Schematic illustration of oxytocin assay used in the study resulting in 3 different fractions: A-plasma, B-IgG-free oxytocin and C-IgG bound oxytocin peptide as summarized in (**b**). For detail see the Materials & Methods section.

**
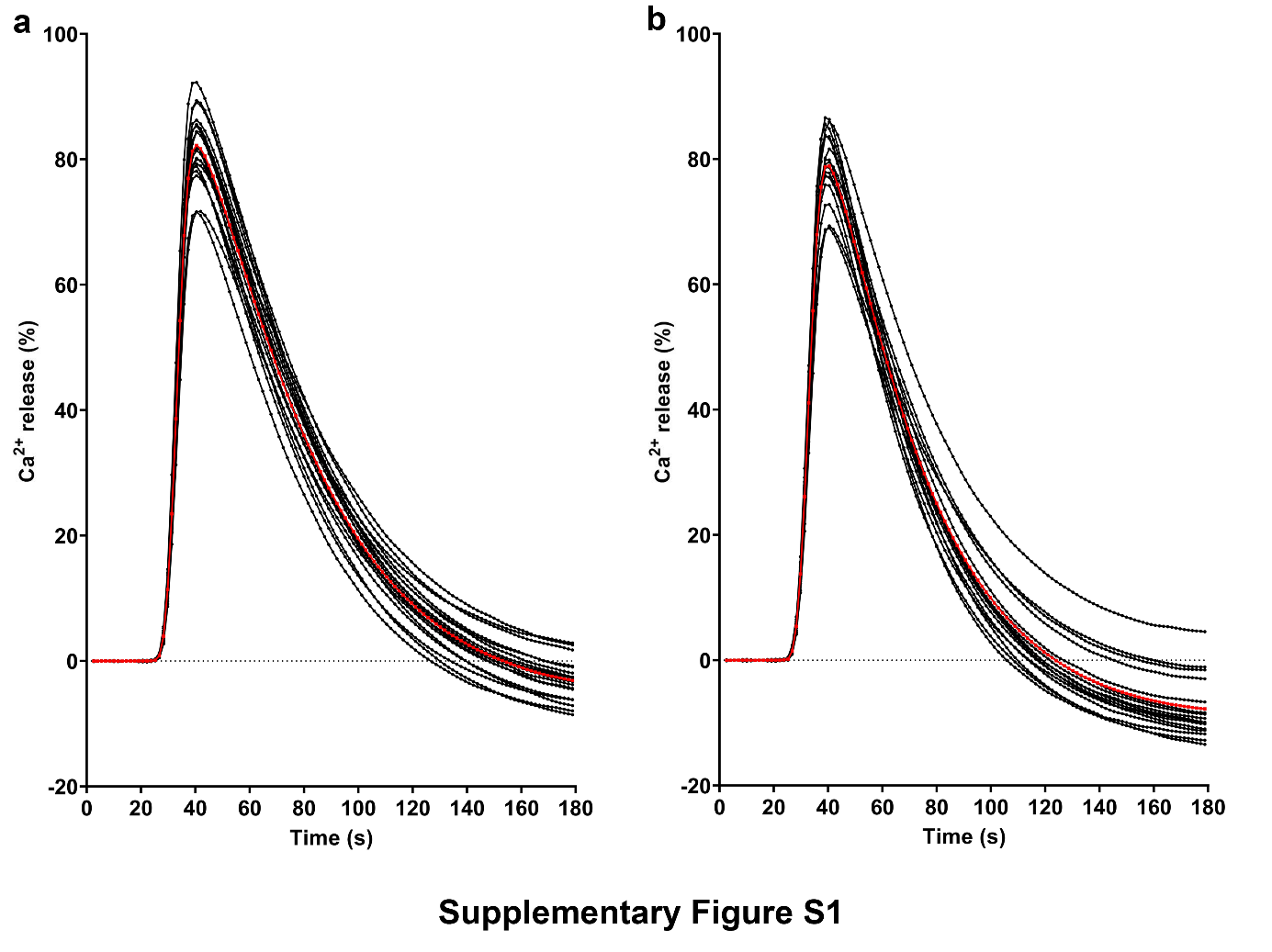
**

**Supplementary Figure 2S. Activation of human OT-R using assay of intracellular Ca^2+^ secretion by OT-R expressing HEK293 cells in vitro.** Intracellular Ca^2+^ secretion by OT-R expressing HEK293 cells in response to oxytocin preincubated with individual IgG from plasma of controls (**a**) and aggressors (**b**). Red lines illustrate the means for each study group.

**
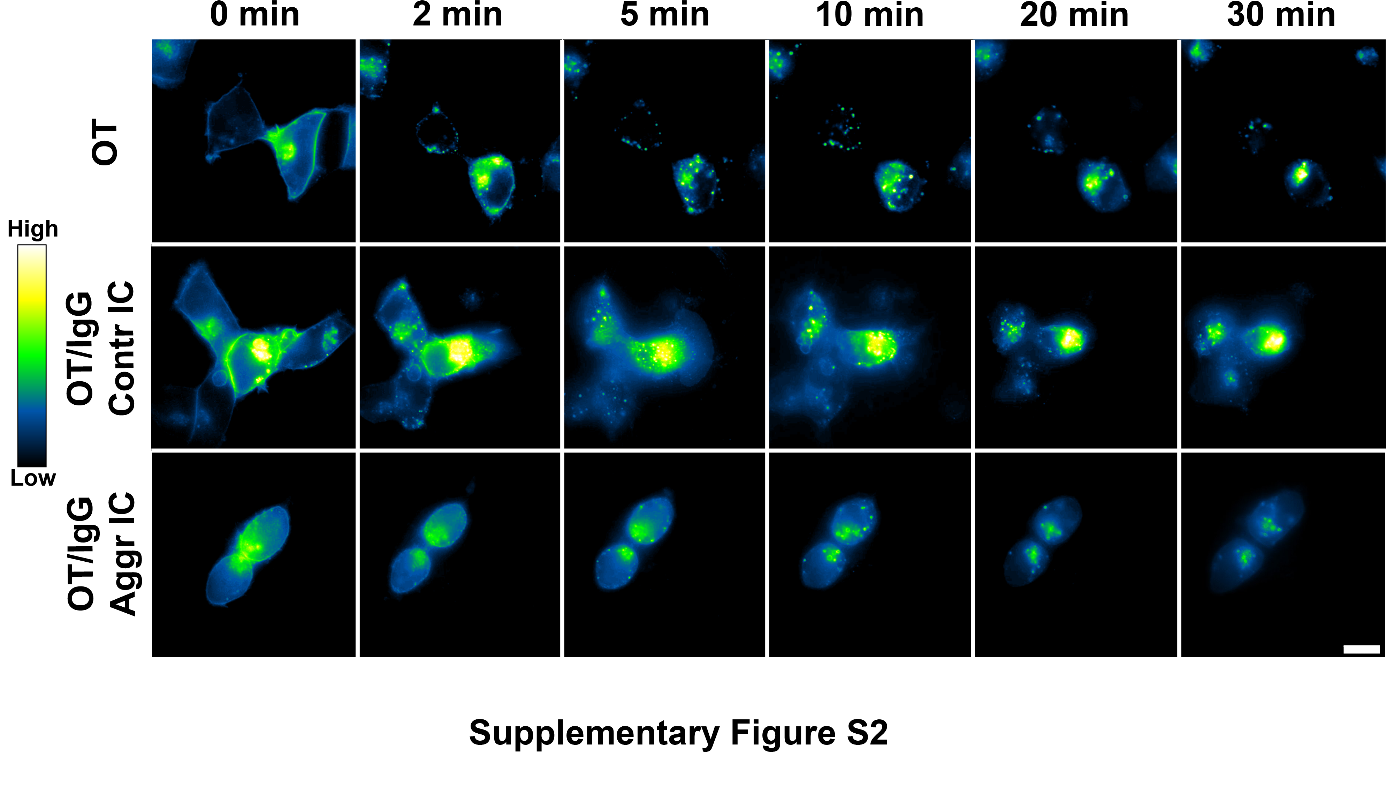
**

**Supplementary Figure 3S. Microscopic visualization of the dynamics of GFP-labeled human OT-R internalization by HEK293 cells in vitro**. Upper line, oxytocin (OT) alone (10^-8^M), 2^nd^ line OT preincubated with IgG (0.5 μM) from controls, and 3^rd^ line OT with IgG from aggressors. Vertical columns correspond to the timing of microscopy before (0 min) and after application of OT alone or OT preincubated with IgG from aggressors or controls. Pseudo-colored fluorescence signal corresponds to the GFP-labeled human OT-R which relative intensity level is shown by a color-map. Scale bar 10 μm.


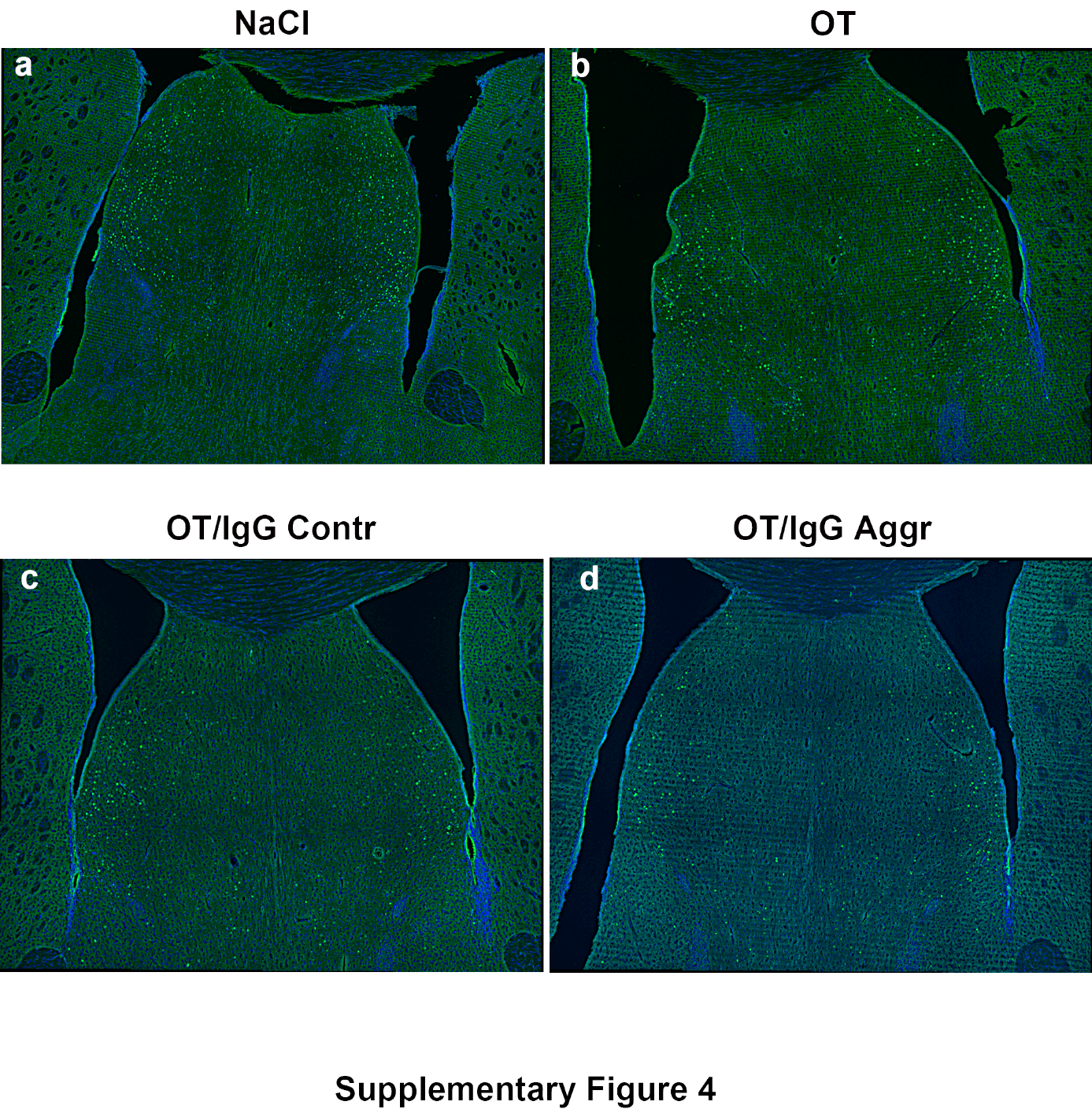


**Supplementary Figure 4S.** Representative microphotographs of immunohistochemical detection of c-fos protein (green) in the mouse septum after the RIT in 4 groups injected with 0.9% NaCl (a), OT (b), OT/IgG Contr IC (c) and OT/IgG Aggr IC (d). Sections were counterstained with DAPI (blue).

**
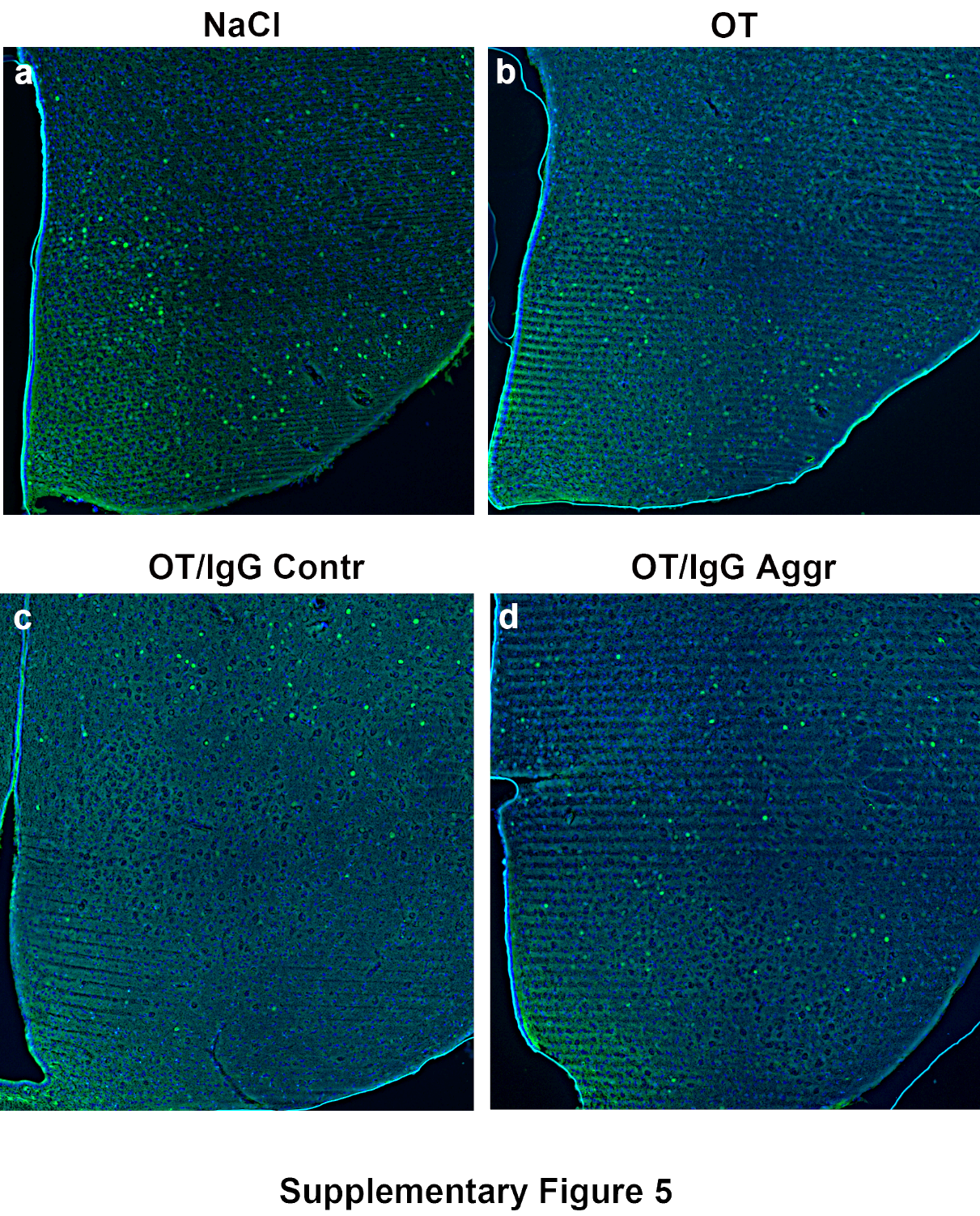
**

**Supplementary Figure 5S.** Representative microphotographs of immunohistochemical detection of c-fos protein (green) in the mouse VMN after the RIT in 4 groups injected with 0.9% NaCl (a), OT (b), OT/IgG Contr IC (c) and OT/IgG Aggr IC (d). Sections were counterstained with DAPI (blue).

**
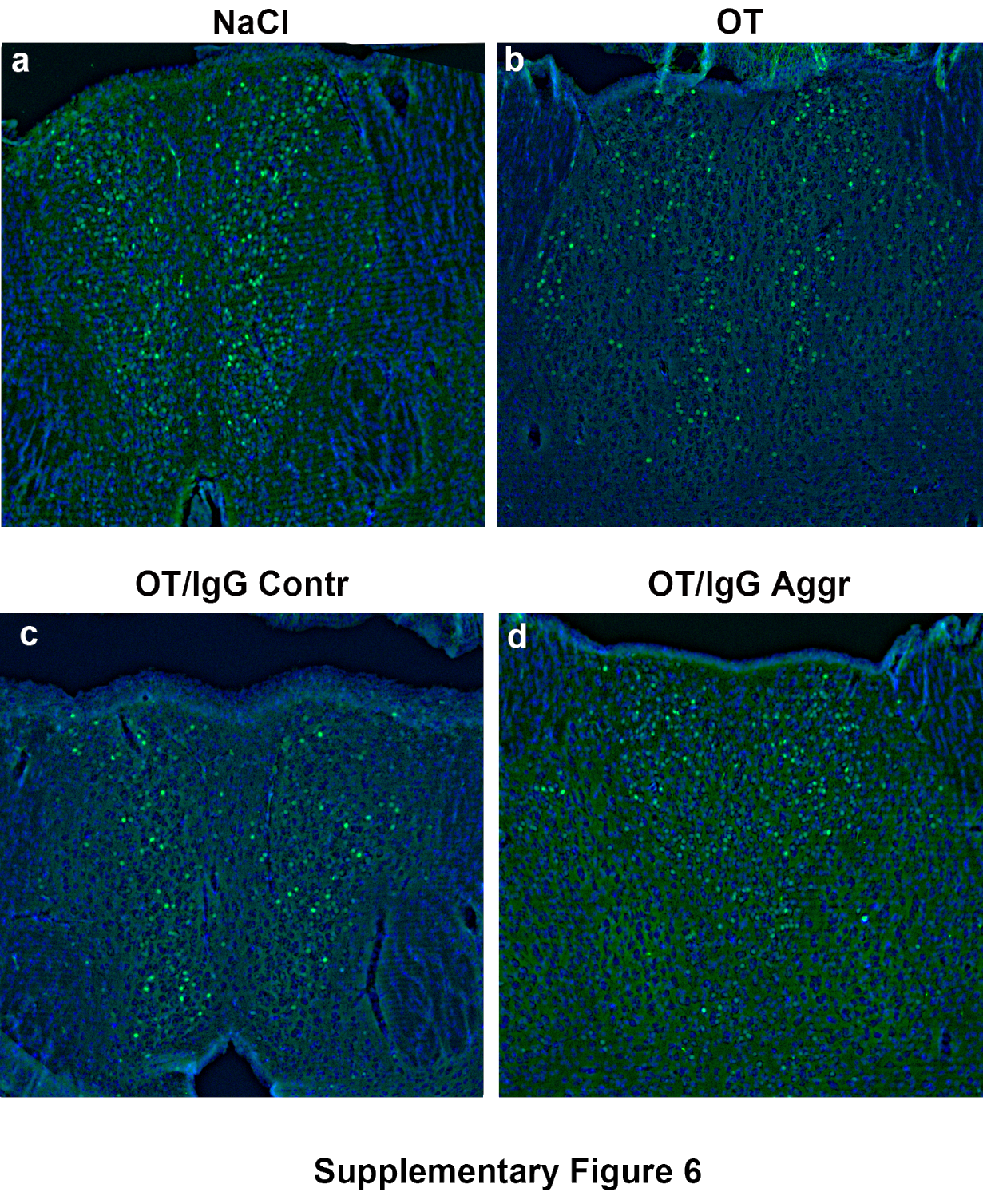
**

**Supplementary Figure 6S.** Representative microphotographs of immunohistochemical detection of c-fos protein (green) in the mouse PVNT after the RIT in 4 groups injected with 0.9% NaCl (a), OT (b), OT/IgG Contr IC (c) and OT/IgG Aggr IC (d). Sections were counterstained with DAPI (blue).

**Supplementary Table 1**

Total and subscale scores (mean + SEM) of the Aggression Questionnaire by Bryant and Smith (BS-rAQ) in male inmates who committed aggressive acts (Inmates) and in a group of men from general population (Controls). Group difference were compared using the Student’s t-test or Mann-Whitney test (MW) showing in bold significant p-value (p<0.05).

|  | Physical aggression | Verbal aggression | Hostility | Anger | BS-rAQ  Total |
| --- | --- | --- | --- | --- | --- |
| Inmates (n=16) | 4.9 + 0.7 | 3.1 + 0.4 | 4.4 + 0.4 | 4.0± 0.7 | 16.4 + 1.8 |
| Controls (n=19) | 1.0 + 0.3 | 2.1 + 0.3 | 1.3 + 0.3 | 1.8 + 0.4 | 6.3 + 0.8 |
| Group difference tests p-values | **p= 0.0002** **MW** | p= 0.07 | **p= 0.0002** **MW** | **p= 0.02 MW** | **p < 0.0001** |

**Supplementary table 2**

Correlations (Pearson’s-“r” ) between BS-rAQ total and subscale scores with concentrations of oxytocin and with levels and properties of oxytocin-reactive IgG in the combined group of men (n=36) which includes inmates who committed aggressive acts (n=16) as well as men from general population (n=19). Significant positive (red) and negative (blue)correlations (*p<0.05, 2-tails) with corresponding p-values are shown in bold.

| Oxytocin (OT)  data | BS-rAQ subscales | | | | | | | Total  BS-rAQ |
| --- | --- | --- | --- | --- | --- | --- | --- | --- |
|  | Hostility | | Verbal  aggression | Physical  aggression | | Anger | |  |
| Oxytocin concentrations | | | | | | | | |
| Plasma OT | **r= 0.36**  **p= 0.03*** | r= -0.12  p= 0.46 | | | r= 0.04  p= 0.79 | | r= -0.01  p= 0.95 | r= 0.06  p= 0.74 |
| Free/unbound  OT | r= -0.15  p= 0.39 | r= -0.21  p=0.22 | | | **r= -0.36**  **p=0.04*** | | r= -0.18  p= 0.29 | **r= -0.37**  **p= 0.03*** |
| Bound OT | r= 0.24  p= 0.17 | r= 0.2  p= 025 | | | r= 0.14  p= 0.42 | | r= 0.07  p= 0.7 | r= 0.17  p= 0.34 |
| Oxytocin IgM & IgG levels | | | | | | | | |
| OT IgM | r= -0.17  p= 0.35 | r= -0.16  p= 0.35 | | | r= -0.27  p= 0.13 | | r= -0.11  p= 0.55 | r= -0.27  p= 0.13 |
| OT IgG free | r= 0.24  p= 0.19 | r= 0.32  p= 0.07 | | | r= 0.26  p= 0.16 | | r= 0.26  p= 0.14 | **r= 0.36**  **p= 0.04*** |
| OT IgG total | r= 0.16  p= 0.37 | r= 0.12  p= 0.53 | | | r= 0.23  p= 0.2 | | r= 0.09  p= 0.6 | r= 0.22  p= 0.22 |
| Oxytocin IgG affinity kinetics | | | | | | | | |
| ka (1/Ms) | r= -0.04  p= 0.8 | r= -0.01  p= 0.9 | | | r= 0.17  p= 0.33 | | r= 0.01  p=0.97 | r= 0.08  p= 0.63 |
| kd (1/s) | r= 0.2  p= 0.24 | r= -0.12  p= 0.48 | | | r= 0.32  p= 0.06 | | r= 0.15  p= 0.38 | r= 0.22  p= 0.19 |
| KD (M) | **r= 0.43**  **p=0.01*** | r=0.01  p= 0.98 | | | r= 0.3  p= 0.08 | | **r= 0.34**  **p= 0.049*** | **r= 0.35**  **p= 0.04*** |
| Oxytocin receptor activation by oxytocin +IgG (Ca^2+^ secretion) | | | | | | | | |
| Ca^2+^ peak value | r= -0.02  p= 0.91 | **r= -0.34**  **p=0.048*** | | | r= -0.28  p= 0.1 | | r= 0.14  p= 0.42 | r= -0.2  p= 0.2 |
| Ca^2+^ AUC | r= -0.323  p= 0.06 | r= -0.29  p= 0.09 | | | **r= -0.37**  **p= 0.027*** | | r= -0.07  p= 0.66 | **r= -0.4**  **p=0.016*** |
